# Drivers of vaccine hesitancy among vulnerable populations in India: a cross-sectional multi-state study

**DOI:** 10.3389/fpubh.2023.1177634

**Published:** 2023-10-09

**Authors:** Parthasarathy Krishnamurthy, Michael S. Mulvey, Kallana Gowda, Meghna Singh, Nitish Kumar Venkatesan, Syama B. Syam, Prerak Shah, Shiv Kumar, Angela Chaudhuri, Raghunathan Narayanan, Abdul Latheef Perne, Aditya Pangaria

**Affiliations:** ^1^Department of Marketing and Entrepreneurship, C. T. Bauer College of Business, University of Houston, Houston, TX, United States; ^2^Telfer School of Management, LIFE Research Institute, University of Ottawa, Ottawa, ON, Canada; ^3^Swasti, Bengaluru, Karnataka, India; ^4^Catalyst Management Services, Bengaluru, India

**Keywords:** vaccine hesitancy, vulnerable populations, fear of vaccination, COVID-19, frontline workers, factor analysis, latent class analysis, multi-level model

## Abstract

**Objectives:**

India’s Covid-19 vaccination campaign engaged frontline workers (FLWs) to encourage vaccination among vulnerable segments of society. The FLWs report encountering a variety of barriers to vaccination and are often unsuccessful despite multiple visits to the same person. This cross-sectional study aims to pinpoint which of these barriers drive vaccine hesitancy among these segments, to help streamline vaccine communication, including FLW training, to better safeguard the population.

**Methods:**

Trained field enumerators contacted 893 individuals from five states across India and collected self-reported assessments of fifteen vaccination barriers (identified through discussions with FLWs), current vaccination status and future vaccination intentions, and covariates (demographics/comorbidities). Factor analysis of the fifteen barriers yielded two factors, one relating to fear of vaccine adverse effects and a second focused on peripheral concerns regarding the vaccine. The covariates significantly associated with current vaccination status were combined under a latent class regime to yield three cluster types (health access, financial strength, and demographics). The primary analysis examined the effect of the two barrier factors, the covariate clusters, and comorbidity, on current vaccination status and future vaccine intentions.

**Results:**

Fear of vaccine adverse effects was the primary driver of vaccine hesitancy; peripheral concerns frequently mentioned by the FLWs had no impact. Although cluster membership and the presence of comorbidities predicted vaccine uptake, neither of them materially altered the effect of fear of vaccine adverse effects with the following exception: fear of adverse effects was not associated with vaccination status among young Muslim men.

**Conclusion:**

Subject to limitations, these results indicate that interventions to decrease vaccine hesitancy should focus primarily on fear associated with vaccines rather than spend resources trying to address peripheral concerns.

## 1. Introduction

The success of vaccination drives is affected by delays in accepting or refusing vaccines. Therefore, it is crucial to understand why people hesitate to get vaccinated, the barriers leading to this hesitancy, and their relative significance. By doing so, we can develop more effective strategies to address this issue. However, unpacking the complexities of this decision can be challenging, especially among socially and economically vulnerable populations, as vaccine hesitancy may also vary spatially across diverse communities and regions. In this paper, we present the findings of a nationwide survey in India that enlisted frontline workers (FLWs) to identify the obstacles that hinder vaccination among vulnerable citizens.

India was one of the worst affected countries by the COVID pandemic, with about 30 million infections and about half a million deaths by August 2021 (1). Like many other countries, India also had the rolled-out vaccination through emergency authorization starting as early as January 16, 2021 (2). The Indian government’s response was one of the world’s most intensive vaccination drives in response to the COVID pandemic. Vaccines were delivered using a multi-stage and phased approach to curtail the spread of the pandemic and minimize its impact. The first stage began with health and frontline workers, extending to the older adults (>60 years old) and comorbid individuals in the second stage, above 45 years old in the third stage, above 18 years old in the fourth stage, 15–18 years old in the fifth stage and has finally reached the stage of vaccine drives for 12–15 years old (3).

This multi-staged phased effort has yielded considerable success, with almost 220 million doses of vaccines administered. However, many challenges hampered the progress of COVID-19 vaccination in the country and amplified disparities across various locations and populations, including concerns about gender and geographical inequities (4–6). One of the major challenges has been vaccine hesitancy (7–9), defined as the refusal or delay in taking the vaccine when available.

A nationwide online survey conducted among the eligible adult population revealed that 37% of the participants were unsure or refused to be vaccinated, and most had one or other concerns about the vaccine, like the rapid development of vaccines, as well as the safety and efficacy of the vaccine (10). The findings from a similar longitudinal survey suggest that the major factors influencing vaccine hesitancy and resistance were concerns about adverse health effects post-vaccination, both major and minor, and lack of clarity about vaccines and their effects on individuals with pre-existing comorbidities (11). Globally, vaccine hesitancy and unwillingness to get vaccinated have been a constant challenge. In the context of a pandemic, addressing hesitancy becomes a critical priority because vaccination is the only effective tool to curtail the spread of this disease when administered to enough individuals (12).

The Indian government vaccination program is carried out by frontline workers who contact the citizens one-on-one and encourage them to get vaccinated. As a result, any obstacles to vaccination usually arise during fieldwork interactions between the frontline worker and the citizen. For this reason, we utilize FLWs as a valuable source of information about the barriers to vaccination in our research approach. This interpersonal approach is a unique feature of the present research.

Frontline workers have reported a variety of barriers to vaccination. Given this assortment, it is vital first to prioritize which barriers to tackle to design FLW training balanced with available time and resources. Frontline healthcare workers may experience physical and mental strain on the job, impacting their effectiveness in addressing barriers. By ranking the barriers by importance, we can ease the burden on FLWs and improve their ability to persuade people to get vaccinated. Additionally, a simplified and personalized approach may alleviate the substantial fatigue and strain associated with this type of work in the field (13, 14).

We assess the strength of the association between vaccine status and barriers identified by FLWs by surveying eligible citizens. Our focus is on vaccine hesitancy among socially and economically vulnerable populations who are hard to reach. Despite having a higher likelihood of not being vaccinated, this group is often underrepresented in research. They are also at greater risk of experiencing acute health and financial impacts if affected by the disease.

It is essential to understand the difference between vaccine uptake and vaccine hesitancy. Vaccine uptake refers to whether a person has been vaccinated, while vaccine hesitancy is a state of indecision and uncertainty before deciding to get vaccinated (15). Vaccine uptake results from both vaccine hesitancy (caused by internal barriers) and external structural factors like vaccine availability. Our research concentrates on examining internal barriers, which is vaccine hesitancy.

In summary, this research aims (a) to catalog the barriers that FLWs encounter when interacting with socially and economically disadvantaged individuals, (b) to determine if the barriers have any commonalities, and (c) to estimate the strength of association between these barriers and vaccine status. To achieve these objectives, we conducted a qualitative pre-study to enlist the barriers to vaccination as reported by FLWs, followed by a quantitative cross-sectional study on the relationship between the barriers and vaccination status. The next section provides details of both studies.

## 2. Materials and methods

The Institutional Review Board of the University of Houston approved the study protocols and informed consent scripts. The qualitative pre-study was conducted as part of the routine operations in which the FLWs periodically meet with program managers to review progress on vaccination rates within their geography. One of the meetings was dedicated to reviewing the barriers. Before that meeting, the FLWs were briefed on the study context and interaction purpose. A similar informed consent form was deployed for the quantitative study and was administered to the respondents by trained enumerators who proceeded with the survey only following consent. Participants in both studies could skip any question, discontinue participation at any stage, and were not paid any monetary or non-monetary incentive to participate. No personally identifiable information was collected.

### 2.1. Qualitative pre-study – identifying frequently encountered barriers

Our first goal was to generate a list of frequently encountered hesitancy barriers to vaccination, as observed by the FLWs. To this end, we conducted guided discussions with the frontline workers who actively encouraged vaccination in the communities.

The program managers initiated the discussions that a member of the author team moderated. The discussions were conducted between March and April 2022 through video conference. We had six video conferences with teams from five states/union territories, Tamil Nadu, Karnataka, Andhra Pradesh, Delhi, and Jharkhand. Each video conference had between 4 and 5 FLWs, in addition to the program manager and a member of the author team who moderated the discussion. Twenty-five FLWs participated in this qualitative study.

The discussions ran about 1–2 h and focused on the following question: “What are some of the major reasons people give for refusing to take the vaccine?” This prompt led to a discussion with each of the FLWs, sharing the barriers they have encountered and bouncing off others’ experiences either in assent or dissent. The moderator’s role was to (a) identify the barriers as and when they were discussed, (b) intervene to clarify, amplify, or qualify any of the barriers, (c) encourage participation by those who were not speaking up, and (d) toward the end of the discussion, summarize the list of the barriers that came up, making any modifications as needed. This discussion resulted in 15 barriers to vaccine hesitancy, as shown in Table 1.

**Table 1:** Measures of vaccination barriers.

Barrier (short form)	Measure
Fear of LT vaccine side effects	I am afraid of long term-side effects of the vaccine
Cannot consume alcohol	I cannot take alcohol/non-veg food before or after the vaccine
COVID is not a big problem	I do not think COVID is such a serious disease
Loss of wages from vaccine side-effects	I am afraid that the side-effects will make me unable to work and earn
Do not trust government	I do not trust government/media information regarding COVID or vaccines
No support for vaccine side-effects	I do not have anyone support me if I have side-effects
Vaccine will worsen health conditions	I am worried that the vaccine will worsen health conditions like BP, Diabetes etc.
Vaccine causes death	I am afraid that the vaccine may cause death
Vaccine is not effective	The vaccine is not effective because people get COVID even after vaccination
Do not like needles/injections	I do not like needles/injections
COVID will not make me sick	Even if I get COVID, I will not get sick
Vaccine can cause infertility	The vaccine can cause infertility
Treating side-effects is costly	Treating vaccine side-effects can be costly
I do not want to become burden to others	I do not want to become burden to others due to side-effects from vaccine
Religion does not permit vaccine	I have religious objections for taking the vaccine

These fifteen barriers (from the guided discussion with frontline workers) were assessed using a 5-point Likert-type scale ranging from strongly disagree (1) to strongly agree (3). The English version is given here, however, the survey was delivered in local languages (Hindi, Kannada, Tamil, and Telugu).

### 2.2. Cross-sectional study: association between barriers and vaccination status

#### 2.2.1. Study design and participants

We conducted a cross-sectional study in India between May and June 2022 to assess the relationship between vaccination barriers (identified by the FLWs in the qualitative pre-study described above) and vaccination status. At the time of the study, the third wave with the omicron variant of COVID had ended, and India had reported 43 million confirmed cases of COVID-19 with 524,000 deaths to the WHO.^1^ The sampling was purposive because we intended to recruit both vaccinated and unvaccinated adults, economically/socially vulnerable populations from diverse geographical backgrounds.

To recruit the participants, we collaborated with community-based organizations that are part of the Covid Action Collaborative (CAC) led by the Catalyst Group. CAC aims to facilitate vaccinations throughout India using a network of over 300 partner organizations. These organizations specialize in serving vulnerable populations by providing health/social services and helping coordinate access to government-run programs. The partner organizations played a critical role in COVID vaccination by training their personnel to become FLWs and sending them out into the community to help increase vaccination among the population.

In consultation with the partners, we identified five states in India, Andhra Pradesh, Jharkhand, Karnataka, Tamil Nadu, and Uttar Pradesh. In addition, within each state, we identified two districts, one with a lower and one with a higher vaccine penetration rate, to increase the chances of having both vaccinated and unvaccinated participants in our final sample.

#### 2.2.2. Questionnaire and measures

The study questionnaire was designed in English in the Qualtrics platform and translated into Telegu, Kannada, Tamil, and Hindi as appropriate. Trained interviewers acted as enumerators for the survey delivery and data collection. Interviewers launched the online survey from their mobile/tablet devices, read the questions to the respondent, and recorded the response.

The principal dependent measures in the study were current covid vaccination status (0, 1, 2, booster) and willingness to take the booster if available (yes, no, or unsure). In addition, we assessed the fifteen barriers identified in discussion with frontline workers using Likert-type 5-point scales (strongly disagree to strongly agree). We followed this up with a set of demographic questions on age, gender (male, female, transgender, other), religion, income source derived from the nature of the occupation (daily, monthly, not working), education, geographic location type (metro/city, town, or village), community background (general, scheduled cast/scheduled tribe, or backward community). We also assessed self-reported comorbidities (high/low blood pressure, heart disease, diabetes, asthma, other). Finally, we assessed questions about the number of government benefits they received, the type of food/social assistance card they had (the ‘ration’ card), and whether they had to support adults in their household.

#### 2.2.3. Statistical analyses

The focus was to identify the most important barriers (among the 15 identified by the FLWs, Table 1) associated with vaccination status. To this end, we conducted two preliminary analyses before the focal analysis.

First, we subjected the set of 15 barriers to factor analysis to identify potential common factors. This analysis resulted in factor scores used to predict vaccination status and future intent.

Second, given that we had a large set of potential additional measures that could impact vaccination status and that these covariates are not necessarily independent, simultaneous inclusion would result in the misspecification of the principal model. Therefore, we subjected the covariates to latent class analysis and used the resulting class membership as proxies for the covariates. The results section presents details of both factor and latent class analysis.

Turning to the primary research goal of the association between barriers and vaccination status, we dichotomized the dependent variable, vaccination status, as “Not vaccinated” (0 doses taken) and “At least one dose taken” (1, 2 doses or booster taken), and predicted this using the factor scores from the factor analysis (detailed later).

Given that the dependent variable was binary, and the individual responses were nested within interviewers, we analyzed the individual responses in a mixed-model framework (PROC GLIMMIX in SAS9.4 M6^®^) with a binary specification for the dependent variable and the interviewer as a random effect in the model. We refer to this as the core hesitancy model because this focuses on how the barriers relate to vaccine hesitancy. We used G*Power3.1 (16) to compute the apriori sample size needed under a logistic regression to detect an odds-ratio of 0.66, assuming a 60% baseline vaccination rate (the rate at that time), an alpha of 0.05, power of 0.95, with a single standardized continuous predictor (factor scores). The needed sample size was 334. That said, we intentionally exceeded the recommended sample size because we expected to test more complex models with multiple predictors.

After estimating the core hesitancy model, we added covariates (from latent class membership) to assess both the robustness of the effect of the barriers on hesitancy and how covariate class membership may modify any of the effects of the barriers. For the additional models, we computed the post-hoc observed power using G*Power since the models involved more predictors than the one used for the *a-priori* sample size.

## 3. Results

Sixteen interviewers completed 893 interviews between May 10 and June 1, 2022. Table 2 displays baseline demographics. We now describe the factor analysis, latent class analysis, and focal analysis of vaccine hesitancy.

**Table 2:** Baseline socio-demographic and situational characteristics.

Variable	Value
Participants (*N*)	893
**Age**	
Mean (SD)	41.0 (14.5)
Range	18.0, 88.0
**Household income, *n* (%)**	
Below 1 Lakh Rupees	793 (89)
Above 1 Lakh Rupees	95 (11)
**Gender, *n* (%)**	
Female	572 (64)
Male	309 (35)
Transgender	8 (1)
**Area, *n* (%)**	
Metro_City_Town	360 (41)
Village	528 (59)
**Community background, *n* (%)**	
GC (general category)	50 (6)
SC/ST (scheduled caste/scheduled tribe)	474 (53)
BC (backward category)	367 (41)
**Primary source of income, *n* (%)**	
Daily wage	488 (55)
Not working	103 (12)
Monthly	298 (34)
**Comorbidities, *n* (%)**	
No	702 (79)
Yes	191 (21)
**Current COVID vax status, *n* (%)**	
Not vaccinated	348 (39)
At least one dose taken	545 (61)
**Education, *n* (%)**	
Not literate	303 (34)
Up to eighth standard	228 (26)
Beyond eighth standard	358 (40)
**Religion, *n* (%)**	
Hindu	554 (62)
Muslim	160 (18)
Other	178 (20)
**Intention to take COVID booster, *n* (%)**	
Yes	455 (52)
No/Unsure	428 (48)

### 3.1. Factor analysis of barrier set

As noted above, the FLWs identified 15 frequently encountered objections/barriers to taking the vaccination in their day-to-day interactions with the end users (Table 1). As noted earlier, our primary goal was to understand how these barriers are associated with vaccination status. A quick scan of the barriers indicated that they may not be entirely independent of each other and treating them as such may induce model specification challenges. For instance, fear of long-term side effects of the vaccine is closer to the fear of exacerbating existing health conditions than it is to trust in media/government. For this reason, we first aimed to assess whether these fifteen barriers can be reduced to a set of common factors, expecting that the common factors will be used as predictors in the core hesitancy model.

To this end, we analyzed the 15 Likert-type items using PROC FACTOR in SAS9.4; the most interpretable model was a 2-factor model (Table 3 for the rotated factor pattern) using promax rotation. Table 4 provides the descriptive statistics associated with the barriers. The first factor comprises barriers associated with concerns about the long and short-term consequences of taking the vaccine. We refer to this factor as “fear of vaccine adverse effects.” The second factor comprises items concerning religious prohibitions, alcohol/meat consumption prohibitions, discounting the nature of COVID infection, discounting the usefulness of the vaccine, etc. We refer to this factor as “peripheral concerns.” Thus, for each participant, we have two factor scores associated with the factors above. The focal analysis used these two factor scores to predict vaccination status (described later).

**Table 3:** Barrier descriptive statistics.

Barrier	Factor	*N*	Mean (SD)
I do not want to become a burden to others^2^	Peripheral concerns	890	3.18 (1.29)
Vaccine is not effective^2^	Peripheral concerns	891	3.08 (1.31)
COVID will not make me sick^2^	Peripheral concerns	891	2.99 (1.29)
No support for vaccine side-effects^2^	Peripheral concerns	891	2.98 (1.26)
COVID is not a big problem		889	2.96 (1.32)
Loss of wages from vaccine side-effects^1^	Fear of AE	891	2.89 (1.32)
Do not trust the government/media^2^	Peripheral concerns	891	2.86 (1.3)
Vaccine will worsen health conditions^1^	Fear of AE	893	2.85 (1.32)
Fear of LT vaccine side effects^1^	Fear of AE	893	2.82 (1.36)
Treating side effects is costly		892	2.80 (1.25)
Cannot consume alcohol^2^	Peripheral concerns	891	2.74 (1.25)
Do not like needles/injections		893	2.63 (1.32)
Vaccine causes death^1^	Fear of AE	891	2.40 (1.26)
Vaccine can cause infertility^1^	Fear of AE	891	2.06 (1.02)
Religion does not permit vaccine		892	1.95 (0.89)

Shortened descriptors are used here to fit the table. Actual measures of the barriers are provided in Table 1. The barriers are sorted on descending magnitude of self-rated agreement (1–5 scale, higher values indicate greater agreement) with the said barrier. The second column shows the factor which each barrier loaded onto (if any; Table 4 for details). Barriers with light shading (second column) load on the “peripheral concerns” factor, and those with darker shade load on the “fear of vaccine adverse effects” factor (hence, fear of AE). Barriers that do not have any factor mentioned in the second column do not load on any factor. Notice that barriers loading on “peripheral concerns 2 rank higher in self-reported agreement (rank 1, 2, 3, 4, 7, and 11) than those loading on factor 1 (6, 8, 9, 13 and 14).

**Table 4:** Rotated factor pattern for the vaccination barriers.

Barrier	Factor 1		Factor 2	
	Fear of vaccine adverse effects		Vaccination-related peripheral concerns	
	Promax	Varimax	Promax	Varimax
Vaccine causes death	74*	72*	−7	6
Loss of wages from vaccine side-effects	72*	71*	−4	8
Fear of LT vaccine side effects	71*	69*	−3	9
Vaccine can cause infertility	53*	54*	8	17
Vaccine will worsen health conditions	50*	52*	15	23
Religion does not permit vaccine	39	39	5	37
Treating side effects is costly	35	39	31	11
COVID will not make me sick	−21	−10	72*	67*
I do not want to become a burden to others	0	10	66*	66*
Vaccine is not effective	16	24	56*	58*
Do not trust the government/media	−3	22	48*	47*
No support for vaccine side-effects	16	4	45*	47*
Cannot consume alcohol	17	23	40*	42*
Do not like needles/injections	28	33	33	38
COVID is not a big problem	12	14	19	20

The above table represents the factor pattern under promax and varimax rotation regimes using a two-factor approach. The asterisk refers to variables which had a factor loading of 40 or higher (automatically generated by PROC FACTOR). We specified 2 to 5 factors under both rotation regimes and found that the two-factor model gave the most interpretable and consistent pattern. Factor scores from the promax rotation were used in modeling hesitancy (Table 6). Shortened barrier descriptors are provided here; the actual barrier items are provided in Table 1.

### 3.2. Latent class analysis

Identifying and targeting subgroups within a population is essential in developing effective and efficient health marketing programs. To this end, we used LatentGOLD version 6.0 (17) to conduct a latent class analysis (LCA) to profile and cluster individuals based on access to healthcare, financial welfare, and socio-demographics using indicators selected on theoretical grounds.

#### 3.2.1. Variables of interest

First, we wanted to test whether Access to Health (AH) impacted individuals’ vaccination status because barriers such as travel distance to the provider and lack of transportation may reduce vaccine status independent of vaccine hesitancy. Costs associated with geographic distance, access to, and modes of transportation can impact vaccination status (18–20). In this study, we investigate patterns in residency (metro/town versus village), distance to health care services (travel time), and mode of transportation to go to the nearest health facility (walk, auto, bus, bicycle) and how they covary with vaccination status.

Second, we examined Financial Welfare (FW) based on patterns in household income, the number of household earners, family support obligations, receipt of government benefits and ration cards, and the ability to receive support from others if needed. In some nations, the receipt of financial benefits is contingent on vaccination (21), and family income is a reason for vaccine hesitancy (22), a plight further complicated for those lacking steady incomes, including migrants and seasonal workers (23). In this study, we consider household income (low, high), receipt of government benefits (count), support of older family members (count), ration card type (none, priority household [PHH], Antyodaya Anna Yojana [AAY] meant for the poorest sections of the population, below poverty line [BPL], and above poverty line [APL]), household earning members (count), and ability to secure the support of others in the community in times of crisis (level).

Third, individuals exhibit heterogeneity concerning age, education, community background, religion, and gender, yet intersections among these Socio-Demographic (SD) variables are typical. For example, research reports higher rates of vaccine refusal among people with a low education level (24), resistance among vaccine-hesitant religious groups (25), and cultural differences based on caste (26). Therefore, this study examines alignments in community background (General Category [GC], scheduled caste/scheduled tribe [SC/ST], and backward category [BC]), level of education, religion, age, and gender.

By reducing many variables into three latent class covariates, we expect to improve the interpretability and actionability of the results and subsequent analyses.

#### 3.2.2. Selecting the number of classes

Next, we ran a latent cluster analysis on the three covariates classes: access to healthcare, financial welfare, and socio-demographics. The analysis involved 873 individuals who completed all the covariate questions. Following conventions, we examined several fit statistics, beginning with BIC, a reliable indicator that rewards model parsimony (27, 28). Lower BICs indicate a better fit. We also examined the Vuong-Lo–Mendell–Rubin (VLMR) adjusted likelihood ratio test and the bootstrapped likelihood ratio (BLR) test (using 500 samples) to assess whether one model is statistically better. Together with theoretical interpretability, these criteria informed our solution choices. Finally, entropy, a diagnostic statistic that indicates a model’s ability to define the classes accurately, is reported but was not used to determine the final class solutions.

These statistics, presented in Table 5, support a three-cluster solution for health accessibility, a four-cluster solution for financial welfare, and a five-cluster solution for demographics. In each case, the recommended model had the best fit based on the lowest BIC values, further supported by the results of the VLR and BLR model comparisons. Each solution (the latent class variable) was more parsimonious than the collection of indicator variables. Also, the entropy index values indicate a good classification of individual cases into clusters. Research team members, including fieldwork leaders, reviewed the best-fitting models to ensure they made sense.

**Table 5:** Latent cluster analysis – model fit evaluation information.

Covariate clusters									
Model	Npar	LL	BIC (LL)	AIC (LL)	*p*-value	Class error	Entropy *R*^2^	BLR *p*-value	VLMR *p*-value
**(A) Health accessibility**									
1-Cluster	8	−2927.4	5909.0	5870.7	0.000	0.00	1.00	–	–
2-Cluster	15	−2684.8	5471.4	5399.7	0.000	0.07	0.77	0.000	0.000
3-Cluster	22	−2655.9	5461.0	5355.7	0.000	0.15	0.65	0.000	0.000
4-Cluster	29	−2641.1	5479.0	5340.3	0.100	0.17	0.70		
5-Cluster	36	−2639.3	5522.7	5350.5	0.007	0.21	0.66		
**(B) Financial welfare**									
1-Cluster	20	−5234.7	10605.0	10509.5	0.000	0.00	1.00	–	–
2-Cluster	32	−5009.7	10236.2	10083.4	0.000	0.00	0.96	0.000	0.000
3-Cluster	44	−4916.8	10131.7	9921.6	0.000	0.06	0.79	0.000	0.000
4-Cluster	56	−4870.5	10120.3	9852.9	0.000	0.08	0.78	0.000	0.000
5-Cluster	68	−4846.3	10153.3	9828.6	0.000	0.15	0.72		
6-Cluster	80	−4827.5	10197.0	9815.1	0.000	0.20	0.70		
**(C) Demographics**									
1-Cluster	11	−6745.6	13565.7	13513.2		0.00	1.00	–	–
2-Cluster	21	−6554.2	13250.7	13150.4	0.000	0.02	0.88	0.000	0.000
3-Cluster	31	−6454.4	13119.0	12970.8	0.000	0.11	0.75	0.000	0.000
4-Cluster	41	−6397.5	13073.0	12877.0	0.000	0.10	0.79	0.000	0.000
5-Cluster	51	−6354.8	13055.3	12811.6	0.000	0.10	0.82	0.000	0.000
6-Cluster	61	−6332.1	13077.8	12786.3	0.013	0.13	0.79		

The headings indicate the number of parameters (Npar) in the fitted model and measures of model fit, including the log-likelihood value (LL), Bayesian information criterion (BIC), Akaike information criterion (AIC), and tests of the bootstrap likelihood ratio (BLR), and Vuong-Lo-Mendell-Rubin adjusted likelihood ratio (VLMR).

#### 3.2.3. Class membership and size

The model class profiles are in the Appendix. Parameter estimates are omitted for space but are available upon request. First, we highlight the top-line findings, focusing on cluster size and distinctive qualities.

Healthcare access (HA) cluster: the LCA model reduced the set of variables to three latent clusters:

- HA Group 1 (31.4%): healthcare is nearby, within walking distance
- HA Group 2 (40.3%): intermediate distance, likely requiring a bus ride, and
- HA Group 3 (28.3%): healthcare is distant, needing auto transportation.

Financial strength (FS) cluster: the financial welfare indicators are reduced to four latent clusters:

- FS Group 1 (46.6%): no government benefits, moderate family support, BPL card
- FS Group 2 (33.6%): some government benefits, greater family support, BPL card
- FS Group 3 (14.3%): some government benefits, no family support, APL, or BPL card
- FS Group 4 (5.5%): higher income, no government benefits or crisis safety net, PHH card.

Socio-demographics (SD) cluster: the indicators reduced to five latent clusters, distinguished as:

- SD Group 1 (29.2%): older adults from a scheduled or tribal caste (SC or SC)
- SD Group 2 (24.1%): younger Hindus from a scheduled or tribal caste (SC or SC)
- SD Group 3 (21.8%): older adults skewed female
- SD Group 4 (18.1%): educated, general category or open (GC or O), and
- SD Group 5 (6.8%): less educated Muslim males from a backward caste (BC).

Individuals’ class membership designations were calculated using the three regression models and saved for subsequent analyses. In summary, each respondent was characterized along three covariate clusters, health access (HA cluster), financial strength (FS cluster), and socio-demographics (SD cluster). Notice that these clusters include external barriers (accessibility to health care facilities) as well as non-hesitancy barriers (gender, religion etc.). This allows us to assess the impact of hesitancy barriers (section 2.3.2.2) on vaccination status while controlling for some non-hesitancy factors.

### 3.3. Models of vaccination status

This section describes the various linear mixed models we specified predicting the two barrier factors (fear of vaccine adverse effects and peripheral concerns) and the covariate cluster membership identified in the latent class analysis (described above). Overall, we specified six models. The statistical significance of the associations between the predictors in the model and vaccination status is summarized in Table 6.

**Table 6:** Linear mixed models of effect of vaccine barrier factors and vaccination status.

Effect	Model 0	Model 1	Model 1a	Model 1b	Model 1c	Model 1d	Model 1e
FearAE	<0.0001	<0.0001	<0.0001	<0.0001	<0.0001	<0.0001	<0.0001
PeriConcerns	0.1956	0.7761	0.867	0.938	0.9316	0.9296	0.4115
Comorbidities		0.0313	0.0387	0.0423	0.0369	0.0564	0.0871
FearAE × Comorbidities		0.1059	0.1096	0.0956	0.1125	0.079	0.0197
PeriConcerns × Comorbidities		0.1741	0.306	0.2576	0.2774	0.3264	0.4677
HealthAccess			0.0012	0.0009	0.0015	0.0006	0.0005
FearAE × HealthAccess				0.9352			
PeriConcerns × HealthAccess				0.1102			
FinancialStrength					0.472		
Demographics						0.0002	0.0001
FearAE × Demographics							0.0065
PeriConcerns × Demographics							0.2585

The above models represent the p-values of the effects (column) on the various modelling regimes (columns). The first two rows represent the two vaccine-related barrier factors, fear of vaccine adverse side effects (FearAE) and peripheral concerns (PeriConcerns) about vaccination. These two factors are always present in every model. Model 0 represents only these two factors. Models 1 through 1e show various covariates being added and removed from the base model, Model 0. Models with highly correlated predictors based on variance inflation factor > 10 using PROC HPREG in SAS9.4® were not estimated.

#### 3.3.1. Core hesitancy model

As noted earlier, the factor analysis indicated that the fifteen barriers arose from two factors, fear of vaccine-related adverse effects and peripheral concerns. These two factor scores were used to predict vaccination status in a linear mixed model framework. We refer to this model as ‘Model 0’.

The results indicated that vaccination status was significantly associated with fear of adverse consequences associated with taking the vaccine, *F*(1, 843) = 67.97, *p* < 0.0001, β = −0.77 (se = 0.09), which translates into an odds ratio of 0.46 (95% CL: 0.39–0.56), indicating that a one-point increase in the factor score for fear of adverse consequences (relative to the mean) is associated with a 54% reduction in the odds of having taken at least one dose. Furthermore, the peripheral concerns factor was not associated with vaccination status, *F*(1, 843) = 1.68, *p* = 0.1956, β = 0.13 (se = 0.10), OR = 1.14 (95% CL: 0.94–1.39).

#### 3.3.2. Additional hesitancy models with covariates

To test the robustness of the effect of fear of vaccine adverse effects on vaccination status, we specified several additional covariates to Model 0, as detailed below.

Model 1 added the presence of comorbidities as a main effect and its interaction with the two factors (fear and peripheral concerns) as additional predictors to Model 0. The results indicated that the presence of comorbidities was significantly associated with vaccination status, *F*(1, 840) = 4.65, *p* < 0.0313, β = −0.44 (se = 0.20), with an odds ratio of 0.64 (95% CL: 0.43–0.96). This indicates that those with comorbidities had 36% lower odds of being vaccinated. Fear of vaccine side effects continued to be a significant predictor of vaccine status, *F*(1, 840) = 53.75, *p* < 0.0001; those with greater fear were less likely to be vaccinated, OR = 0.51 (95% CL: 0.42–0.63). No other effects were statistically significant predictors of vaccination status.

Model 1a added health access cluster membership (easy, moderate, and difficult access) as a covariate. In this model, health access was a significant predictor of vaccination status, *F*(1, 829) = 6.79, *p* = 0.0012; those in the difficult access cluster had 60% lower odds of being vaccinated compared to those who belonged to the easy health access cluster, OR = 0.39 (95% CL: 0.24–0.65). Although the moderate health access cluster had directionally lower odds of vaccination than the easy access cluster, OR = 0.69 (95% CL: 0.45–1.05), the difference did not reach statistical significance. As with Model 0 and Model 1, vaccine status was significantly associated with fear of vaccine side effects *F* (1, 829) = 46.18, *p* < 0.0001, OR = 0.53 (95% CL: 0.43 to 0.66), and with comorbidities, *F*(1, 829) = 4.29, *p* = 0.0387, OR = 0.65 (95% CL: 0.43 to 0.98). No other effects were statistically significant predictors of vaccination status. The key takeaway from Model 1a is that the health access cluster is a significant predictor of vaccination status along with fear of vaccine side effects and the presence of comorbidities.

Model 1b added the interaction between health access cluster membership and the two barrier factors, fear of side effects and peripheral concerns, to Model 1a. However, neither of these interactions was statistically significant, and none of the other effects from Model 1a changed substantively.

Model 1c added financial strength cluster membership as a covariate and removed the interactions involving health access cluster membership. Financial strength cluster membership was not significantly associated with vaccination status. However, the previously significant effects, fear of adverse effects, comorbidities, and health access cluster membership, continued to remain significant predictors of vaccination status.

Model 1d added demographic cluster membership as a covariate and removed the financial strength cluster from Model 1c. The results showed that demographic cluster is significantly associated with vaccination status; compared to educated respondents in the general category, the group comprising women with higher age, education, and membership in the general category had 40% lower odds of being vaccinated, and Muslim men had 82% lower odds of being vaccinated. In addition, the previously significant effects from Model 1c, fear of vaccine side effects and health access cluster, remained significant. However, the presence of comorbidities was no longer statistically significant, *p* = 0.0564. Non-significant effects from Model 1c remained non-significant.

Finally, Model 1e added interaction between demographic cluster membership and the two barrier factors, fear of vaccine side effects and peripheral concerns, to Model 1d. In addition to preserving the main effect of fear of vaccine side effects, *F* (1, 809) = 26.43, *p* < 0.0001, OR = 0.59 (95% CL: 0.42–0.83), health access cluster membership, *F* (1, 809) =7.65, *p* = 0.0005, and demographic cluster, *F* (1,809) = 5.95, *p* = 0.0001, we observed an interaction between demographic cluster and fear of vaccine side effects, *F* (1, 809) = 3.36, *p* = 0.0097. Specifically, the negative effect of fear on vaccination status was attenuated for older women and neutralized for young Muslims. This is suggestive of the possibility that there might be other forces than fear of vaccine side effects that account for low vaccine adoption in these groups. In the next section, we discuss the implication of these findings.

## 4. Discussion

This research aimed to understand the relative impact of various vaccine hesitancy barriers on vaccination status. Understanding and tackling vaccine hesitancy is crucial because it delays or stops people from getting the protection they need and prevents the achievement of herd immunity. We focused on hesitancy among the vulnerable sections of society because they need more health and financial protection.

The COVID vaccination drive in India relied heavily upon frontline workers (FLWs) who sought to vaccinate hundreds of millions of people through interpersonal interactions with them, either one-on-one or in small groups. This direct interaction with hesitant citizens, attentive listening, adept questioning, and astute observation of nonverbal cues empowers frontline workers (FLWs) to uncover latent concerns and uncertainties among unvaccinated citizens. As such, FLWs are an excellent source of information on vaccination barriers. This proactive approach facilitates early issue detection, enabling organizers to swiftly address emerging challenges and effectively curb potential escalations.

Our empirical strategy included a preliminary qualitative study where we had discussions with FLWs to identify the barriers they face. From the discussions, we distilled fifteen barriers (Table 1). Our primary goal was to assess the relative impact of the FLW-identified barriers on vaccination status. Vaccination status. We conducted a cross-sectional study and contacted nearly 900 participants from ten districts across five states with varied cultural and geographic features and vaccine penetration levels. Before studying their effect on hesitancy, we first investigated whether the barriers had common underlying factors. Factor analysis revealed that fear of side effects (fear of death, fear of lost wages, fear of long-term side effects, fear of infertility, and exacerbation of comorbidities) and peripheral concerns (discounting the effectiveness of the vaccine, discounting the concerns about COVID, religious concerns, concerns about alcohol/meat consumption, etc.) explained the fifteen barriers best.

The results indicated that fear of side effects was the principal and robust driver of hesitancy. Although peripheral concerns came up frequently in the FLW discussions and were often rated as generally more important (rank of 1, 2, 3, 4, 7, and 11) than fear-related barriers (rank of 6, 8, 9, 13, and 14), the latter consistently and strongly predicted vaccine hesitancy. This finding reveals that asking people which barriers are more important does not necessarily correlate with what holds sway regarding vaccine hesitancy. Peripheral concerns may appear more important than they were (they were not important) in predicting vaccination status.

We tested the impact of potential covariates that may modify this core finding with a host of variables under a latent class clustering regime to group participants into three clusters based on their health access, financial status, and social-demographic traits. The advantage of this approach is that it is statistically efficient (allows estimation of the clusters) and potentially insightful for the types of messages different class clusters should receive. In addition, we considered comorbidities as an independent covariate. Finally, the covariates’ effects were assessed in various regimes (Table 6). The results indicated the following. First, the effect of fear of vaccine side effects as the primary driver of vaccine hesitancy was robust to covariates in the model. Second, the health access cluster was consistently associated with vaccine status; those far away were significantly less likely to be vaccinated. Third, mere membership in certain social-demographic clusters (older women and Muslim men) was associated with lower vaccination.

These findings have specific implications for what the communication should focus on and to whom. While there has been extraordinary effort spent in mobilizing the FLWs to help vaccinate the population, the training for the FLWs has focused primarily on the clinical protocol, such as maintaining the integrity of the vaccine (cold chain), proper sterilization at the point of vaccination, etc. While this training is crucial to maintain supply, it does little to address vaccine hesitancy, which, as noted earlier, is the uncertainty/delay/deferral when the vaccine is available for the citizen. This approach requires additional training for the FLWs to help handle the objections they encounter. Specifically, our research finds the fear of adverse consequences of vaccination as the primary hesitancy driver. Therefore, FLW training should address vaccination-related fears and place lesser emphasis on peripheral concerns that seem interesting but are empirically unrelated to hesitancy (peripheral concerns, Table 4). Given the significant fatigue and potential mental health challenges the FLWs experience Field (13, 14) when encouraging citizens to vaccinate, this training assumes additional importance.

Although our study was in the context of COVID vaccination, the findings’ implications could go beyond COVID vaccines. Fear of adverse consequences of vaccination is not limited to COVID vaccines; it has the potential to apply to all vaccines. There is already a disturbing downturn in the non-COVID vaccination among children. As it stands, there is a disturbing trend of lower routine immunizations in the post-pandemic phase (29), partly due to the elevated media attention on concerns about vaccine safety. For this reason, it is imperative that the findings from this study be examined in the context of non-COVID vaccines and followed up with studies that point to ways of addressing vaccine-related fears in general.

In addition, vaccine supply considerations are a good candidate for focus in areas with low health access. Moreover, places with vaccine shortages may consider age-specific transmission risks (30) and vaccine allocation strategies to reduce deaths and new infections (31). Finally, regarding the social-demographic clusters at risk for low vaccination, our study does not have a specific prescription. It was not designed to assess underlying reasons and therefore warrants further study.

In this regard, the models presented in this research focus on vaccination status as the dependent variable. As noted in the methods section, we also measured future intentions regarding the booster. However, we did not present the analysis in the interest of expositional simplicity. The analysis of future intentions is ongoing, and the preliminary results indicate that fear of adverse consequences of vaccination continues to be the principal driver of whether people intend to vaccinate.

While the presented findings are noteworthy, there are some limitations to consider and opportunities for further exploration. Firstly, the sample is limited to five states and communities the organizational partners serve, making it difficult to generalize to the wider population. Secondly, the study was conducted from May to July 2022, so the findings may not reflect the current situation regarding barriers to vaccination. However, it is worth noting that the finding that vaccine hesitancy is related to adverse effects is likely applicable to all vaccines, not just COVID-19. Nevertheless, more research is needed to confirm this.

Moreover, the finding that hesitancy is related to adverse effects of vaccination probably applies to all vaccines, but the presented results cannot assert that without further research. The fear of vaccine side effects is notable as it may apply to hesitancy towards all vaccines, not just COVID vaccines. The study’s cross-sectional nature limits the results to association rather than causation, suggesting that future studies should use an appropriate methodological framework to examine the potential causal relationship between fear of vaccine side effects and vaccine hesitancy.

Additionally, the study did not address the timing of vaccination adoption, which may provide insights into how to increase the speed of adoption, which is crucial in managing infectious diseases. Finally, the drivers of hesitancy are conceptualized as unchanging over time, which may not be the case. A longitudinal study may reveal important shifts that predict a change in hesitancy, which requires further research. This holds particular significance considering the potential for ‘pandemic fatigue,’ a phenomenon in which individuals progressively diminish their vigilance and precautionary measures against infections over time (32). This not only sustains the prevalence of the virus but also amplifies the risk of emergence for vaccine-resistant mutant strains (33).

Lastly, prospective studies could explore the potential and constraints of engaging Frontline Workers (FLWs) within vaccination initiatives. Our proposed approach acknowledges the capability of FLWs to actively listen, generate innovative insights, and tailor their responses to align with the evolving needs of citizens. This concept draws inspiration from the adaptive selling paradigm in marketing, which underscores the benefits of empowering employees for agile customer interactions (34, 35). Notably, this competency is not solely contingent on personnel selection but can be effectively nurtured through comprehensive training interventions (36). There remains a considerable research gap in public health, necessitating exploring strategies to influence employee adaptability and cultivate an organizational ethos that fosters empowerment, ultimately contributing to an enhanced customer experience.

## Data availability statement

The raw data supporting the conclusions of this article will be made available by the authors, without undue reservation.

## Ethics statement

The Institutional Review Board of the University of Houston approved the study protocols and informed consent scripts. Before the first phase of the data collection, FLWs were briefed on the study context and interaction purpose. A similar informed consent form was deployed for the study's second phase by trained enumerators who proceeded with the survey only after receiving consent. Both sets of participants could skip any question, discontinue the survey at any stage, and were not paid any monetary or non-monetary incentive to participate. No personally identifiable information was collected.

## Author contributions

Study design: PK, with major contributions from NV, KG, and PS, and critical contributions from SK, AC, and RN. Data collection: KG, AbP, NV, and PS, with critical contributions from PK. Data analysis and methods write-up: PK and MM (latent class analysis). First draft of manuscript: PK with major contributions from MS and SS. Critical revisions: PK, MM, NV, MS, SS, KG, and AdP. All authors contributed to the article and approved the submitted version.

## Footnotes

^1^ https://covid19.who.int/region/searo/country/in
