# Supplementary material for: Drivers of vaccine hesitancy among vulnerable populations in India: a cross-sectional multi-state study
Source: Front Public Health. 2023 Oct 9;11:1177634. doi: 10.3389/fpubh.2023.1177634 (PMC10600374; doi:10.3389/fpubh.2023.1177634)
Supplement: Supplementary file 1 [file Data_Sheet_1.docx]

**Appendix – Cluster response percentages within variables**

**(A) Health Access Cluster profile**

|  |  | |  | |  |  |
| --- | --- | --- | --- | --- | --- | --- |
|  | **Cluster1** | **Cluster2** | | **Cluster3** | | |
|  | **Easy accessibility** | **Moderate accessibility** | | **Low accessibility** | | |
| Cluster Size | 0.31 | 0.40 | | 0.28 | | |
|  |  |  | |  | | |
| Indicators |  |  | |  | | |
|  |  |  | |  | | |
| **Area** |  |  | |  | | |
| Metro/City/Town | 0.79 | 0.34 | | 0.07 | | |
| Village | 0.21 | 0.66 | | 0.93 | | |
|  |  |  | |  | | |
| **Distance to Health Facility** |  |  | |  | | |
| Less_than_15_minutes | 0.82 | 0.05 | | 0.00 | | |
| 15 to 30_minutes | 0.17 | 0.60 | | 0.07 | | |
| 30 to 60_minutes | 0.00 | 0.33 | | 0.49 | | |
| More than 60 minutes. | 0.00 | 0.02 | | 0.44 | | |
|  |  |  | |  | | |
| **Commute to Health Facility** |  |  | |  | | |
| Walk | 0.60 | 0.08 | | 0.01 | | |
| Auto | 0.27 | 0.44 | | 0.82 | | |
| Bus | 0.03 | 0.29 | | 0.11 | | |
| Bicycle | 0.08 | 0.15 | | 0.06 | | |
| Other | 0.02 | 0.03 | | 0.00 | | |

| **(B) Financial Strength Cluster profile** | | | | | | | |
| --- | --- | --- | --- | --- | --- | --- | --- |
|  |  |  | |  | |  | |
|  | **Cluster1** | | **Cluster2** | | **Cluster3** | | **Cluster4** |
|  | **Baseline** | | **AAY, +supports +gov ben** | | **BPL.APL  -income** | | **PHH. +income**  **-crisis** |
| Cluster Size | 0.47 | | 0.34 | | 0.14 | | 0.06 |
|  |  | |  | |  | |  |
| Indicators |  | |  | |  | |  |
|  |  | |  | |  | |  |
| **Ration Card** |  | |  | |  | |  |
| PHH (Priority House Hold) | 0.02 | | 0.00 | | 0.00 | | 0.92 |
| AAY (Antyodaya Anna Yojana) | 0.08 | | 0.02 | | 0.00 | | 0.00 |
| BPL (Below Poverty Line) | 0.64 | | 0.72 | | 0.44 | | 0.02 |
| APL (Above Poverty Line) | 0.03 | | 0.23 | | 0.51 | | 0.04 |
| No ration card | 0.21 | | 0.02 | | 0.05 | | 0.02 |
|  |  | |  | |  | |  |
| **Household Income** |  | |  | |  | |  |
| Above 1 Lakh | 0.07 | | 0.06 | | 0.00 | | 0.98 |
| Below 1 Lakh | 0.93 | | 0.94 | | 1.00 | | 0.02 |
|  |  | |  | |  | |  |
| **Need to Support Family** |  | |  | |  | |  |
| Never | 0.14 | | 0.03 | | 0.81 | | 0.83 |
| Sometimes | 0.63 | | 0.46 | | 0.18 | | 0.17 |
| Always | 0.23 | | 0.51 | | 0.00 | | 0.00 |
|  |  | |  | |  | |  |
| **Government Benefit** |  | |  | |  | |  |
| 0 | 0.93 | | 0.03 | | 0.04 | | 1.00 |
| 1 to 2 | 0.07 | | 0.89 | | 0.90 | | 0.00 |
| 3 to 4 | 0.00 | | 0.04 | | 0.04 | | 0.00 |
| 5 or above | 0.00 | | 0.04 | | 0.03 | | 0.00 |
|  |  | |  | |  | |  |
| **Others to Reach out in Crisis** |  | |  | |  | |  |
| Always | 0.17 | | 0.23 | | 0.14 | | 0.00 |
| Often | 0.35 | | 0.40 | | 0.32 | | 0.00 |
| Don't know | 0.05 | | 0.05 | | 0.05 | | 0.00 |
| Rarely | 0.25 | | 0.20 | | 0.26 | | 0.06 |
| Never | 0.19 | | 0.13 | | 0.22 | | 0.93 |
|  |  | |  | |  | |  |
| **Earning Members in Family** |  | |  | |  | |  |
| 1 | 0.50 | | 0.43 | | 0.52 | | 0.67 |
| 2 | 0.40 | | 0.43 | | 0.39 | | 0.29 |
| 3 | 0.07 | | 0.09 | | 0.06 | | 0.03 |
| 4 | 0.02 | | 0.04 | | 0.02 | | 0.01 |
| 5 | 0.01 | | 0.01 | | 0.01 | | 0.00 |

| **(C) Socio-Demographic Cluster Profile** | | | | | | | | | |
| --- | --- | --- | --- | --- | --- | --- | --- | --- | --- |
|  |  |  | |  | |  | |  | |
|  | **Cluster1** | | **Cluster2** | | **Cluster3** | | **Cluster4** | | **Cluster5** |
|  | **Older SC/ST** | | **Young Hindu or Other** | | **Older female** | | **Educated GC** | | **Young Muslim Male BC** |
| Cluster Size | 0.29 | | 0.24 | | 0.22 | | 0.18 | | 0.07 |
|  |  | |  | |  | |  | |  |
| Indicators |  | |  | |  | |  | |  |
|  |  | |  | |  | |  | |  |
| **Age** |  | |  | |  | |  | |  |
| Mean | 49.00 | | 31.55 | | 51.74 | | 34.26 | | 25.74 |
|  |  | |  | |  | |  | |  |
| **Religion** |  | |  | |  | |  | |  |
| Hindu | 0.55 | | 0.74 | | 0.63 | | 0.77 | | 0.07 |
| Muslim | 0.01 | | 0.00 | | 0.34 | | 0.23 | | 0.93 |
| Other | 0.44 | | 0.26 | | 0.03 | | 0.00 | | 0.00 |
|  |  | |  | |  | |  | |  |
| **Education** |  | |  | |  | |  | |  |
| Illiterate | 0.58 | | 0.02 | | 0.51 | | 0.00 | | 0.82 |
| Upto8thStandard | 0.36 | | 0.22 | | 0.40 | | 0.03 | | 0.16 |
| Beyond8thStandard | 0.07 | | 0.76 | | 0.09 | | 0.97 | | 0.01 |
|  |  | |  | |  | |  | |  |
| **Gender** |  | |  | |  | |  | |  |
| Female | 0.71 | | 0.68 | | 0.67 | | 0.50 | | 0.48 |
| Male | 0.28 | | 0.31 | | 0.33 | | 0.47 | | 0.52 |
| Transgender | 0.01 | | 0.01 | | 0.00 | | 0.03 | | 0.00 |
|  |  | |  | |  | |  | |  |
| **Community Background** |  | |  | |  | |  | |  |
| BC | 0.01 | | 0.04 | | 0.88 | | 0.80 | | 0.98 |
| General Category Open | 0.00 | | 0.00 | | 0.10 | | 0.18 | | 0.01 |
| SC/ST | 0.99 | | 0.96 | | 0.02 | | 0.02 | | 0.00 |

BC refers to Backward Category

GC refers to General Category

SC/ST refers to Scheduled Caste/Scheduled Tribe
